# Stem Cell-Derived Regulatory T Cells for Therapeutic Use in Arthritis

**DOI:** 10.16966/2470-1025.119

**Published:** 2016-06-27

**Authors:** Jianxun Song

**Affiliations:** Department of Microbiology and Immunology, The Pennsylvania State University College of Medicine, Hershey, Pennsylvania, USA

**Keywords:** Pluripotent stem cells, Autoimmune arthritis, Stem cells

## Abstract

Pluripotent stem cells (PSCs) can be utilized to obtain a renewable source of healthy regulatory T cells (T_regs_) to treat autoimmune arthritis as they have the ability to produce almost all cell types in the body, including T_regs_. However, the right conditions for the development of antigen (Ag)-specific T_regs_ from PSCs (*i.e.*, PSC-T_regs_) remain unknown. An ongoing project will determine the mechanisms underlying the Ag-specific PSC-T_reg_ treatments that aim to modulate tolerance in autoimmune arthritis. The knowledge gained from these studies will provide new insights into cell-based therapies in autoimmune arthritis, and advance the understanding of fundamental mechanisms underlying T_reg_ differentiation.

Regulatory T cells (T_regs_) are an integral component of the normal immune system and contribute to the maintenance of peripheral tolerance. T_regs_ can down-regulate immune responses and are essential for immune homeostasis. They can act as key effectors in preventing and treating rheumatoid arthritis (RA) [1,2].

Hematopoietic stem cell (HSC)-derived hematopoietic progenitors migrate into the thymus and develop into different types of T cells. The transcription factors Aire (largely expressed in thymic medullary epithelial cells - mTECs) and FoxP3 have key functions in clonal deletion and T_reg_ selection [3]. There are links between Aire expression, FoxP3 upregulation and T_reg_ selection; Aire deficiency affects the negative selection of self-reactive T cells, and FoxP3 controls the development and function of the naturally occurring T_regs_ (nT_regs_) [4]. Our laboratory has shown the development of stable T_regs_ from CD4^+^ T cells by over-expressing FoxP3 and bcl-xL [5].

Recent advances in the use of large-scale *in vitro* expansion of T_regs_ followed by *in vivo* re-infusion of these cells raises the possibility that this strategy may be successfully utilized for the treatment of *rheumatoid arthritis (RA)* [6]. Although polyclonally expanded populations of T_regs_ exhibit suppressive activity, antigen (Ag)-specific T_regs_ are more efficient at suppressing local autoimmune disorders such as RA, type-1 diabetes (T1D), inflammatory bowel diseases (IBD), allergic reactions and graft-versus- host disease (GVHD) [7–11]. In addition, tissue/organ-associated T_reg_ targeting stabilizes FoxP3 expression and avoids induction of a potentially detrimental systemic immunosuppression [12,13]. For T_reg_-based immunotherapy, *in vitro* generation of tissue/organ (e.g., synovium)-associated and non-terminally differentiated effector T_regs_ for *in vivo* reinfusion is an optimal approach. However, current methodologies are limited in terms of the capacity to generate, isolate, and expand a sufficient quantity of such T_regs_ from patients for therapeutic interventions.

A number of challenges exist in T_reg_-based immunotherapy:

## First

Only low numbers of T_regs_ can be harvested from the peripheral blood mononuclear cells (PBMCs). CD4 and CD25 have been used to isolate T_regs_ for ex vivo expansion. CD4^+^CD25^+^ T cells are not homogenous and contain both T_regs_ and conventional effector T cells (T_effs_). Current expansion protocols activate both T_regs_ and T_effs_, and because it takes a longer time for T_regs_ to enter the S phase of cell cycle, T_effs_ outgrow T_regs_ [14]. In addition, T_regs_ can lose suppressive activity after repetitive stimulation with α-CD3 plus α--CD28 antibodies (Abs) with or without rIL-2 *in vitro*.

## Second

No approach to date has demonstrated the capacity to isolate the entire T_reg_ population with 100% specificity from patients (the current clinical approach). Even FoxP3 or more recently Eos, a transcriptional factor that is considered the gold standard for identification of T_regs_, is expressed transiently in some activated non-regulatory human T cells [15], highlighting the difficulty in both identifying and isolating a pure T_reg_ population. The adoptive transfer of non-regulatory T_effs_ with T_regs_ has a potential to worsen autoimmune diseases.

## Third

Gene transduction of CD4^+^ T cells from PBMCs with Ag-specific T cell receptor (TCR) [16] or chimeric Ag receptor (CAR) [17] and/or TCR with FoxP3 elicits the generation of suppressive T cell populations [8] and overcomes the hurdle of the limited numbers of Ag-specific T cells. However, the engineered T_regs_ express endogenous and exogenous polyclonal TCRs, which reduce their therapeutic potential (the current experimental approach). Also, TCR mispairing is a concern with regards to the safety of TCR gene-transferred T_regs_ for clinical use, because the formation of new heterodimers of TCR can induce immunopathology [18]. Therefore, there is a need to improve this strategy and generate monoclonal T_regs_.

## Fourth

The differentiation state of T_regs_ is inversely related to their capacity to proliferate and persist. The “right” T_regs_ resist terminal differentiation, maintain high replicative potential (e.g., expression of common- γ chain- γ_c_, CD132), are less prone to apoptosis (e.g., low expression of PD-1), and have the ability to respond to homeostatic cytokines [19], which facilitates their survival. In addition, the “right” T_regs_ express high levels of molecules that facilitate their homing to lymph nodes (LNs), such as CD62L and CC-chemokine receptors (e.g., CCR4, CCR7), and maintain stability or plasticity under certain inflammatory conditions. Furthermore, after an effective immune response, the “right” T_regs_ persist and provide protective immunity.

## Fifth

Because there are too few cells, harvesting sufficient numbers of tissue-associated T_regs_ from PBMCs for TCR gene transduction can be problematic.

Taken together, strong arguments support the development of T_reg_-based therapies in autoimmune arthritis using engineered T_regs_. While clinical trials show safety, feasibility, and potential therapeutic activity of T_reg_-based therapies using this approach, concerns about autoimmunity due to cross-reactivity with healthy tissues remains a major safety issue [20,21]. In addition, genetically modified T_regs_ using current approaches are usually intermediate or later effector T_regs_ [22], which only have short-term persistence *in vivo*.

To date, pluripotent stem cells (PSCs) are the only source available to generate a high number of the “right” T_regs_ [23,24]. Human induced PSCs (iPSCs) can be easily generated from patients’ somatic cells by transduction of various transcription factors and exhibit characteristics identical to those of embryonic stem cells (ESCs) [25]. Many genetic methods as well as protein-based approaches have been developed to produce iPSCs with potentially reduced risks, including that of immunogenicity and tumorigenicity [26]. Because of the plasticity and the potential for an unlimited capacity for self-renewal, iPSCs have high potential for advancing the field of cell-based therapies.

Our laboratory was the first to show that the development of Ag-specific iPSC-CTLs or iPSC-T_regs_ can be used for cell-based therapies of cancers and autoimmune disorders [23,24,27–30] other groups reported similar results[31–33]. We demonstrated that genetically modified iPSCs with Ag-specific TCR and the transcriptional factor FoxP3, followed by differentiation driven by Notch signaling can enable iPSCs to pass hematopoietic and T lineage differentiation checkpoints, resulting in the development of Ag-specific CD4^+^T_regs_. We have developed a novel system to generate stable Ag-specific iPSC-T_regs_. Our ongoing studies will validate this system and provide new insights into the methodologies and mechanistic requirements for efficient development of inflamed tissue-associated iPSC-T_regs_. Once such strategies become available, there is potential to facilitate the generation of tolerance for autoimmune arthritis. Thus, important advances towards T_reg_-based immunotherapy in autoimmune arthritis are anticipated from the proposed studies.

PSCs are exposed to a number of signals responsible for their progression. Although the exact signals are not fully understood, part of the mechanism known to be critical for directing T-cell fate occurs via Notch signaling. The Notch is evolutionarily conserved; regulating cell fate decisions in a number of cell and tissue types. Ligand binding by members of the Jagged or Delta-like (DL) families results in the proteolytic cleavage and release of the intracellular fragment of the Notch heterodimer. Translocation to the nucleus then allows for its regulation of gene expression. Notch-1, specifically, is critical for the establishment of T-cell fate. The loss of function results in the blockade of T cell development and enhanced B cell production, while over-expression results in the blockade of B cell lymphopoiesis and leads to the generation of T cells [34]. However, the intracellular signaling pathways by which Notch signaling regulates the differentiation of Ag-specific PSC-T_regs_ remain unknown. PSCs co-cultured on a monolayer of the bone marrow (BM) stromal cell line OP9 cells transfected with the Notch ligand DL1 or 4 exhibits the ability to differentiate into most hematopoietic lineages and T cells [31]. Our studies will determine the critical regulations of Hes1 [35], Runx1 [36], and surviving [37] by Notch signaling during the development of autoAg-specific PSC-T_regs_.

Although Ag-specific human iPSC-T_regs_ may have promising therapeutic effects in cell-based therapies, their efficiency is limited by the need to generate a large number of such cells using complex and expensive *in vitro* differentiation. In addition, the lengthy duration for generating human iPSCs may limit their use in individualized therapies. Alternatively, we will perform cell-based therapies using the TCR/FoxP3 gene-transduced iPSCs, which can differentiate into auto Ag-specific iPSC-T_regs_ *in vivo* and suppress autoimmune arthritis. We will perform arthritis induction before or after the adoptive transfer of the gene-transduced iPSCs. We will inject Notch agonists or recombinant cytokines (e.g., rIL-7, rFlt3L) to boost *in vivo* development of auto Ag-specific iPSC-T_regs_.

In summary, a current roadblock to progress in the field is the lack of an efficient system to generate the “right” autoAg-specific T_regs_ that could be used for cell-based therapies in autoimmune arthritis. We propose the use of PSC-T_regs_ to address this limitation, allowing derivation of a large number of stable autoAg-specific PSC-T_regs_ for cell-based therapies. Development of such an approach provides an important step toward personalized therapies for autoimmune arthritis.
